# Clinical and Economic Outcomes Associated With Musculoskeletal Care in an Integrated Advanced Primary Care Model: Controlled Cohort Analysis

**DOI:** 10.2196/76794

**Published:** 2025-10-07

**Authors:** Courtenay J Stewart, Dena M Bravata, Michael T Nelson, Esha Datta, Raj Behal

**Affiliations:** 1 Clinical Effectiveness Amazon One Medical San Francisco, CA United States; 2 Stanford Clinical Excellence Research Center Stanford, CA United States; 3 Stanford Center for Primary Care and Outcomes Research Stanford, CA United States; 4 Agile Physical Therapy Palo Alto, CA United States

**Keywords:** advanced primary care, delivery of health care, health care costs, integrated, multidisciplinary care, musculoskeletal diseases, pain, patient satisfaction, physical therapy specialty, telemedicine

## Abstract

**Background:**

Health care costs in the United States are skyrocketing, with commercial spending increasing 7.7% between 2022 and 2023. Musculoskeletal conditions affect more than one-third of US adults and account for over US $300 billion in total medical spending, more than any other chronic condition. Employers bear a disproportionate burden of these costs, both because they pay for the care of employees and their families with musculoskeletal conditions and because musculoskeletal pain is the second leading cause of workplace absenteeism, accounting for approximately 290 million lost workdays annually. Tele-physical therapy (TPT) solutions can be an effective alternative to in-person physical therapy (PT) and, especially when provided early in the course of care, have the potential to reduce employer-sponsored health care spending.

**Objective:**

We sought to evaluate the effects of a proactive musculoskeletal treatment approach—TPT integrated into advanced primary care—on patient access, changes in functional status, and employer cost.

**Methods:**

We performed a retrospective analysis of participants (>13 years old) seen by TPT integrated with primary care compared to a risk-adjusted, nationally matched cohort of patients receiving PT. The studied intervention had five key elements: (1) a multidisciplinary team, (2) a musculoskeletal toolkit for primary care physicians, (3) a peer-to-peer musculoskeletal expert opinion portal, (4) a shared technology platform, and (5) musculoskeletal educational rounds. We collected participants’ access to both primary care and PT and compared participants’ functional status at baseline and at the end of their course of PT to risk-adjusted Focus on Therapeutic Outcomes controls, providers’ assessments of participants’ progress with PT, participants’ satisfaction with their TPT, and costs of care.

**Results:**

We evaluated 1563 participants whose average age was 42.8 (SD 10.4) years. Of these, 586 (37.5%) identified as female, 574 (36.7%) as White, 182 (11.6%) as Asian, and 19 (1.2%) as Black or African American. Their presenting complaints included shoulder pain (282/1563, 18%), knee pain (250/1563, 16%), and low back pain (187/1563, 11.96%). The mean time to TPT appointment was 7.6 (SD 5) days. On average, TPT patients required 5.4 (SD 2.7) visits to symptom resolution, compared to 6.5 (SD 5.5) visits for controls (a 17% reduction) and 10.3 (SD 1.55) predicted visits from risk-adjusted benchmarks, resulting in US $193 to US $1411 in savings per injury per patient. Recovery, defined as patients either meeting, mostly meeting, or on track to meet expectations, was achieved for 461/473 (97.5%) participants for whom it was assessed. Overall participant satisfaction was high, with a net promoter score for PTs of 97.

**Conclusions:**

TPT integrated with advanced primary care was associated with greater functional improvement in 17% fewer visits compared to usual care. This model holds considerable promise for addressing the escalating musculoskeletal costs of US commercially insured populations.

## Introduction

Health care costs in the United States are skyrocketing, with commercial spending increasing 7.7% between 2002 and 2023 [1]. Musculoskeletal conditions affect more than one-third of US adults [2] and account for over US $300 billion in total medical spending—more than any other chronic condition [3]. Employers bear a disproportionate burden of these costs [1], both because they pay for the care of employees and their families with musculoskeletal conditions and because musculoskeletal pain is the second leading cause of workplace absenteeism [4]. In the United States, the majority of health care spending for musculoskeletal conditions among working-age adults (20-64 years) is funded by employer-sponsored health insurance. For example, in 2016, 57.2% of the US $134.5 billion spent on low back and neck pain and 56.4% of the US $129.8 billion spent on other musculoskeletal disorders were paid by employer-sponsored plans [3]. Beyond these direct health care costs, employers incur substantial indirect costs, including sickness absence, lost productivity, and disability payments [4]. Studies in both the United States and Europe have shown that musculoskeletal disorders are the leading cause of work disability, absenteeism, and reduced on-the-job productivity, with total costs (including lost productivity) estimated to account for 290 million lost workdays annually [5-7].

Timely assessment and early use of physical therapy (PT) can improve musculoskeletal functional status and health outcomes and avoid unnecessary spending for musculoskeletal disorders, including fewer PT visits, advanced imaging, opioid prescriptions, and specialist visits [8-12]. Employers have implemented solutions that provide their employees with rapid access to high-quality musculoskeletal care [13,14]. These include centers of excellence, near-site and on-site clinics with musculoskeletal offerings, tele-physical therapy (TPT), musculoskeletal care coordination services, and digital musculoskeletal therapeutics [15-17]. Especially during the pandemic, the use of digital interventions dramatically increased [18]. Early evidence for these interventions held promise for both increasing early access to musculoskeletal care and decreasing costs of avoidable specialist visits, imaging tests, and surgical procedures [10,19,20]. A recent review of employer-sponsored musculoskeletal solutions found that app-based exercise therapy solutions may be appropriate for patients with lower acuity needs and that TPT solutions can be an effective alternative to in-person PT and have the potential to reduce health care spending [14].

Prior research has demonstrated the value of in-person musculoskeletal care integrated with primary care and of stand-alone TPT [21]. For example, embedding PTs within primary care clinics has been shown to improve referral completion rates and reduce time to care for musculoskeletal complaints [22]. Multiple systematic reviews have demonstrated that TPT is noninferior to in-person care for pain, function, and patient satisfaction in a variety of musculoskeletal disorders [23-25]. However, there are no published studies evaluating TPT integrated within an advanced primary care practice. The objectives of this study were to describe and evaluate the effects of a care model that includes TPT integrated into a hybrid (telemedicine and in-office) primary care system on access to musculoskeletal care, functional status outcomes, patient experience, and cost of care compared to patients cared for in the community.

## Methods

### Participants and Controls

We retrospectively analyzed data from a cohort of adolescent and adult participants (age greater than 13 years) with musculoskeletal concerns seen by a One Medical TPT between January 1, 2021, and December 7, 2023. Because we sought to understand clinical improvement over time, we excluded participants who had only 1 TPT visit or did not complete both baseline and final clinical assessments (as these participants would not have both pretreatment and posttreatment data).

We compared the clinical outcomes of the patients seen in TPT to all eligible controls in the Focus on Therapeutic Outcomes (FOTO) database [26]. FOTO provides outcome measurement software for rehabilitation therapists. When collecting patient-reported outcome data in the FOTO system, patients enter their own data and outcome scores are automatically tabulated. FOTO has the largest available dataset on outpatient physical medicine outcomes in the United States and uses a regression analysis that accounts for the patient characteristics and health factors as predictors of functional status improvement [27,28]. FOTO Controls were matched to participants on age, sex, body part, predicted functional score change, predicted number of visits, acuity as assessed by the number of days from onset of the treated condition, type of insurance, BMI, and number of medical comorbidities (although they were not controlled on specific conditions). The FOTO risk-adjustment model was used to calculate a predicted discharge functional score and the predicted number of visits to achieve this functional score. This predicted functional score and number of visits are shared with the patient and treating physical therapist, so they can track how their progress compares to patients just like them.

### Data Collection

We assessed participants’ access to both primary care and PT, compared participants’ functional status at baseline and at the end of their course of PT to risk-adjusted FOTO controls, providers’ assessments of participants’ progress with PT, participants’ satisfaction with their TPT, and costs of care.

#### Timing of Data Collection

Participant data were collected at baseline, during the course of PT, and at their final appointment. Baseline assessments were sent to participants through electronic messaging within their electronic health record during the week prior to their initial evaluation. Ongoing assessments of participants’ status were collected at the discretion of the provider, with a goal of collecting assessment data at either the fourth appointment or within 30 days of the initial examination. Final assessments were sent following the patient’s final PT appointment. Patients lost to follow-up were contacted within 2 weeks to determine the reason for self-discharge and to attempt to collect a final status assessment.

#### Access to Care

We assessed access to both primary care and TPT. We measured the time to see a primary care provider using the time to the third next available appointment or the average number of business days for patients to book a provider’s third next available appointment, which is a commonly used measure of health care access [10]. We measured the time to start PT from the date a scheduler received confirmation of insurance approval for PT to the participant’s initial appointment, which was selected based on the patient’s availability. Data to measure access times were extracted from the electronic health record from August 3, 2022, to December 7, 2023.

#### Patient-Reported Outcomes

Demographics and comorbid conditions were extracted from the electronic health record. Participants were asked to complete the well-validated FOTO instrument [29]. FOTO uses the functional status score, where 0 indicates low function and 100 indicates high function. Participants completed functional status questionnaires before treatment, during treatment, and at discharge.

#### Provider-Reported Outcomes

Providers categorized participants’ progress with PT as meeting the goals that were jointly established at the start of therapy in 3 levels: mostly meeting expectations, on track, or off track. Provider-reported participant progress was extracted from the electronic health record for the 473 participants seen between February 16, 2021, and December 7, 2023. We excluded participants from this analysis who were lost to follow-up or transferred out of TPT prior to when the provider-reported participant progress could be assessed.

#### Costs of TPT

Direct costs of PT care were calculated from insurance claims for direct PT billing in 2023 for patients receiving care at One Medical in California [30].

#### Patient Satisfaction

Participants were sent the following net promoter score question at the end of every visit through email: “How likely is it that you would recommend the provider you saw to a friend or colleague? 0: Not at all likely, 10: Extremely likely.”

### Intervention

The intervention was provided by physical therapists in the One Medical integrated care system. Physical therapists work 1:1 with patients during 45-minute synchronous video visits. The same physical therapist conducts the initial visit and the subsequent follow-up visits to ensure continuity of care. Physical therapists were educated about telemedicine care through a required 5-hour telehealth training course and had to pass a competency examination. Additionally, each therapist received two 1:1 training sessions with a senior therapist, each of which included a mock patient encounter. Care quality is maintained through regular review of FOTO outcomes by a senior therapist, clinical rounds education, regular case discussion, and chart reviews to assess for evidence-based guideline adherence.

### Musculoskeletal Care in an Integrated Advanced Primary Care Model

PT is integrated into an advanced primary care model with 5 key elements: multidisciplinary team, musculoskeletal toolkit for primary care physicians (PCPs), peer-to-peer musculoskeletal expert opinion portal, shared technology platform, and musculoskeletal educational rounds. Table 1 provides an overview of how this model of care differs from stand-alone telemedicine musculoskeletal care and usual musculoskeletal care.

**Table 1:** A comparison of the components of 3 alternative approaches to the provision of musculoskeletal (MSK) care in the United States: primary care with integrated tele-physical therapy (TPT), stand-alone telemedicine musculoskeletal care, and usual musculoskeletal care.

MSK care component	Primary care with integrated TPT	Stand-alone telemedicine MSK care	Usual MSK care
Synchronous telemedicine PT^a^	Always^b^	Sometimes^c^	Sometimes
Rapid or direct access to MSK care	Always	Always	—^d^
MSK care integrated with behavioral health care	Always	Always	—
Medication management	Always	—	Sometimes
Direct messaging between patients and care team	Always	Sometimes	Sometimes
PCP^e^-prescribed at-home exercise program (alternative to PT)	Always	Sometimes	—
Peer-to-peer MSK expert consultation for PCPs	Always	—	—
Evidence-based MSK guidance for PCPs in EHR^f^	Always	—	—
Care coordination and navigation	Always	—	—
Specialist referrals (eg, neurosurgery and orthopedics)	Always	—	Always

^a^PT: physical therapy.

^b^Indicates core component of this care model, typically always available.

^c^Indicates that this component is sometimes available as part of this care model.

^d^Indicates that this is not available in this care model.

^e^Primary care physician.

^f^EHR: electronic health record.

#### Multidisciplinary Team

Patients have access to 4 specialties: primary care (delivered by physicians, physician assistants, and nurse practitioners with training in family practice, internal medicine, geriatrics, and sports medicine), behavioral health (delivered by psychologists, licensed clinical social workers, licensed mental health counselors, and marriage and family therapists), health coaching (delivered by board-certified health and wellness coaches, many of whom have additional certification in nutrition counseling), and musculoskeletal rehabilitation care (delivered by physical therapists).

Participants can receive specialized musculoskeletal care in a variety of ways: (1) participants can book a synchronous video visit or in-office visit with their primary care provider or (2) access on-demand care through in-app messaging or video chat. Primary care providers evaluate patient concerns and develop an assessment and individualized treatment plan. This may include medications, in-office lab tests, imaging orders, and referral to specialty care, procedures, or PT (either telemedicine within the integrated team or external in-person care) if indicated. Once enrolled in TPT, the TPT shares progress notes and coordinates care for emerging needs (such as in-person care for manual procedures or injections, adding on behavioral health assessment, or needed imaging) with the PCP. Common contraindications to TPT include impaired balance without caregiver support and hands-on postoperative care.

**Figure 1:**
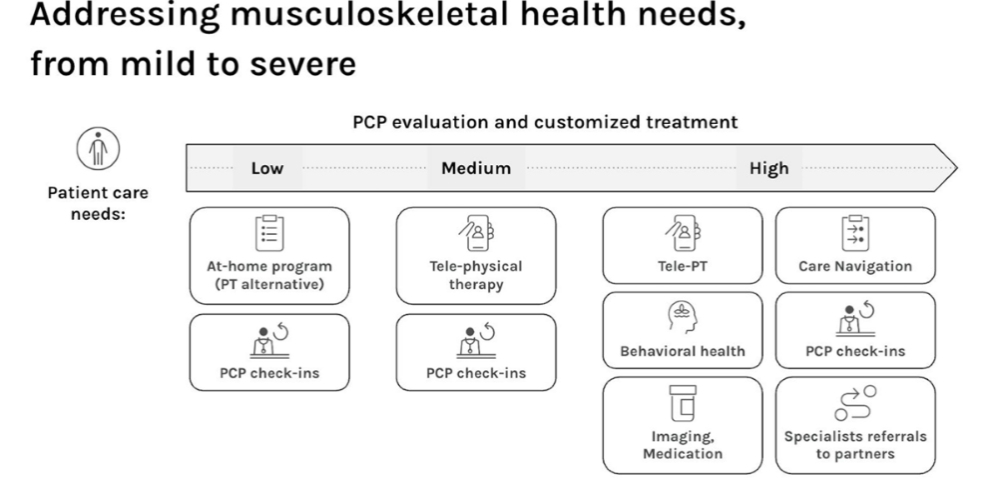
Musculoskeletal care (including tele-physical therapy, imaging, and referral to specialists) matched to patient needs as determined in primary care. Primary care physician (PCP) evaluation and customized treatment are supported by the musculoskeletal toolkit, which includes evidence-based musculoskeletal guidance in the electronic medical record and a peer-to-peer musculoskeletal expert opinion portal. PT: physical therapy.

Depending on patients’ needs, other clinical services are available that are integrated with the core multidisciplinary team (Figure 1). Patients with higher-acuity care needs have access to the Complex Care Team, which provides care navigators and nurses for patients requiring multiple referrals, intensive medication management, or hospitalization. Patients with selected chronic conditions (eg, obesity and diabetes) are eligible for disease management services, which include a team-based approach using remote patient monitoring (eg, activity tracking and glucose monitoring), education, laboratory testing, and behavior change interventions. Each One Medical market also includes local health system partners who provide specialty care (eg, orthopedics, oncology, and neurosurgery).

#### Musculoskeletal Toolkit

PCPs have access to a musculoskeletal toolkit that drives five key objectives: (1) Ensure consistent, evidence-based musculoskeletal recommendations by all PCPs across the organization. This consistency includes the use of common language regarding chronic pain and recovery by all team members [31]. (2) Enable PCPs to match the patient to the best treatments for them early in their care (eg, PT before specialist referrals for many patients, starting patients with unaddressed psychosocial factors on both PT and behavioral health treatment or stratified care [32], and referral for imaging or procedures before PT when indicated). (3) Encourage patient buy-in ahead of PT by harnessing the trusted, longitudinal PCP relationship. PCPs are trained to use shared decision-making around the initial pain education and treatment selection, which includes discussion of the patient’s pain management goals and treatment expectations. (4) Educate patients on the latest guidelines for the care of their specific condition [16], including whether their condition requires surgery or imaging, the appropriate use of manual therapy in conjunction with other therapies, and the importance of the role of PT. (5) Provide anticipatory guidance for painful conditions, as PT and movement may increase pain and soreness in the short term. The PCP discussion focuses on helping the patient understand the expected pain trajectory ahead of treatment to encourage PT adherence.

The toolkit is available at the point of care to reduce PCP burden around decision-making and challenging patient-provider discussions. The toolkit consists of care guidelines, ready-to-prescribe at-home treatment programs, and ongoing education. Musculoskeletal care guidelines are evidence-based, embedded in the electronic health record, and include guidance on common physical examination maneuvers, diagnostic recommendations, conservative care, when to consider imaging or specialty referrals, and best practices for communicating with patients (Multimedia Appendix 1). Ready-to-prescribe at-home treatment programs (Multimedia Appendix 2) are available for the most common musculoskeletal conditions seen in primary care. They are created by musculoskeletal experts (eg, physical therapists, sports medicine physicians, and physiatrists), which include biopsychosocial education and therapeutic exercises. These materials focus on functional status and include aspects of behavioral health education and fundamentals of pain science. These are typically used as a PT alternative or bridge before PT and help ensure that education and exercises are delivered as quickly as possible.

#### Peer-to-Peer Musculoskeletal Expert Opinion Portal

PCPs have access to a messaging portal with One Medical musculoskeletal specialists (eg, physical medicine and rehabilitation physicians, sports medicine physicians, and physical therapists) for quick curbside consults. This service uses Slack technology to enable PCPs to ask questions and learn from the responses being provided to their own questions (typically the same day) and those of their colleagues. PCPs post questions before, during, or after a clinical encounter with a patient. The nature of these questions includes “When to refer to PT vs specialist referral?” “What is your interpretation of this imaging study?” “This patient is not getting better as expected—what is the best next step?”

#### Shared Technology Platform

The integrated electronic health record facilitates team-based care. Specifically, internal providers can and do easily refer to each other and provide real-time questions, patient updates, and handoff details. Commonly, PTs will identify new issues that require PCP attention (such as a new medication prescription), and patients can easily receive this care in-house. The PCP delivers and coordinates care for patients simultaneously enrolled in behavioral health and PT for chronic musculoskeletal pain. Providers can coordinate their care plans using a real-time provider messaging forum. Moreover, patients are spared having to repeatedly provide historical information that is key to the care of their musculoskeletal issues, including history of trauma, chronic pain diagnoses, and recurrent injuries in the same body part, given that this information is available to all members of the care team.

Patients have an app, which they use to schedule appointments, manage prescriptions, message their care team, speak with a provider through video visits 24/7, and access their care plan and health records. All patient-provider messages are visible to all members of the care team. The platform is available as a native app on iOS and Android devices and as a web app on personal computers.

#### Musculoskeletal Educational Rounds

All members of the care team participate in musculoskeletal educational rounds, which cover a range of primary care topics, including pain, injury, and common musculoskeletal care. Some musculoskeletal educational rounds are developed by the internal One Medical clinical learning team (eg, updates on the evidence of mental health interventions for chronic pain); others are presented by visiting lecturers (eg, specialists from affiliated academic referral centers) and internal musculoskeletal experts.

### Analysis

We used univariate analyses to describe the demographics of patients, measures of their access to care, patient-reported outcomes, and provider-reported outcomes. We considered analyses with *P*<.05 to be statistically significant. For the clinical outcomes, we conducted a retrospective matched cohort analysis comparing patients receiving TPT integrated with primary care to controls in the FOTO dataset. Specifically, we compared the number of visits and functional status for the TPT patients with those predicted from FOTO and with FOTO controls. We computed a 2-sided *t* test on patients’ functional status and number of visits residuals (residuals are the FOTO-predicted value minus what was actually observed for a patient).

### Ethical Considerations

All data used in this analysis were routinely collected from patients receiving care at One Medical clinics and were deidentified before analysis. The WIRB-Copernicus Group Institutional Review Board deemed this protocol exempt (Protocol OM.001; October 5, 2023). Patients received no compensation for participation in this evaluation.

## Results

### Participant Characteristics

**Figure 2:**
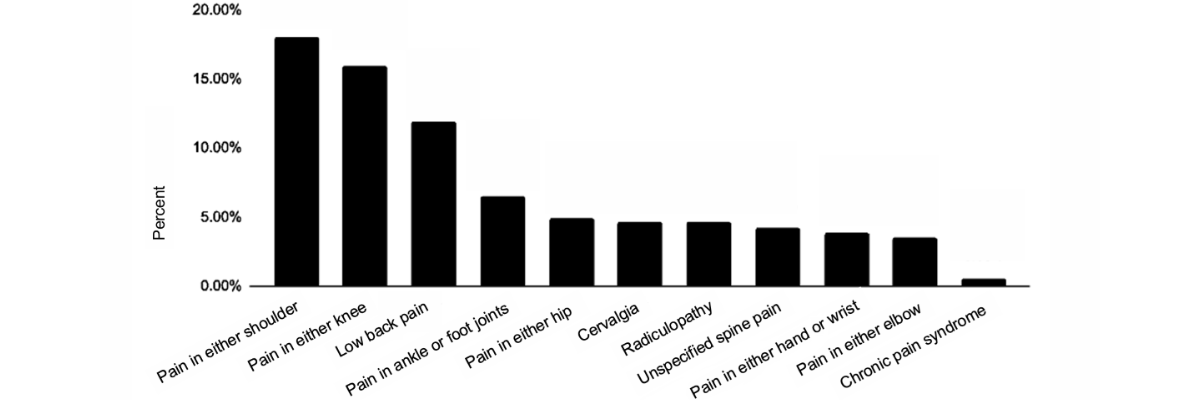
Prevalence of the primary musculoskeletal complaint at presentation in primary care by affected body part (shown as percentage of total population, N=1563).

The average age of the 1563 participants was 42.8 (SD 10.4) years. Of these, 586 (37.5%) identified as female, 574 (36.7%) as White, 182 (11.6%) as Asian, and 19 (1.2%) as Black or African American. On average, participants were overweight (mean BMI 26.3, SD 5.4 kg/m^2^; Table 2). Their most common musculoskeletal conditions at baseline were shoulder pain (282/1563, 18%), knee pain (250/1563, 16%), and low back pain (187/1563, 12%; Figure 2).

**Table 2:** Participant characteristics (N=1563).

Characteristics		Value
**Age (years), mean (SD)**		42.8 (10.4)
Age groups (years), n (%)		
	16-30	139 (8.9)
	31-45	786 (50.3)
	46-60	538 (34.4)
	>60	100 (6.4)
**Sex, n (%)**		
	Male	731 (46.7)
	Female	586 (37.5)
	Not reported	242 (15.5)
	Nonbinary	4 (0.3)
**BMI (kg/m** ^**2**^ **), mean (SD)**		26.3 (5.4)
BMI categories, n (%)		
	None reported	683 (43.7)
	<18.5 (underweight)	21 (1.3)
	18.5-25 (normal weight)	414 (26.5)
	25-30 (overweight)	300 (19.2)
	>30 (obese)	145 (9.3)
**Race and ethnicity, n (%)**		
	Not reported	712 (45.6)
	White	574 (36.7)
	Asian	182 (11.6)
	Multiple races	42 (2.7)
	Other	24 (1.5)
	Black or African American	19 (1.2)
	Middle Eastern or North African	8 (0.5)
	Native American or Alaska Native	2 (0.1)

### Access to Care

We measured time to access both primary care and PT. The mean third next available appointment for PCPs was 6.6 (SD 8.2) days. On average, time to see a PT (after referral and insurance checks are complete) was 7.6 (SD 5) days. Early PT is typically defined as care initiation within 30 days of the index health care visit [10].

### Health Services Use

Of the 1563 patients in TPT, the comorbidities for which they received primary care included anxiety (n=435, 27.8%), depression (n=280, 17.9%), overweight or obesity (n=259, 16.6%), hypertension (n=177, 11.3%), and chronic kidney disease (n=41, 3%). On average, PCPs sought 2.7 peer-to-peer musculoskeletal expert opinion consults per week.

After referral and initiating TPT, most patients (1257/1375, 91.4%) were able to be cared for in PT alone (excluding 188 patients lost to follow-up). Small proportions of patients required additional care: 57/1375 (4.2%) patients were referred back to their PCP for further evaluations or medication management (eg, imaging and specialty consults) and 77/1563 (5%) were transferred to in-person PT. Notably, 188/1563 (12%) patients were lost to follow-up (stopped responding to physical therapist or scheduler messages or failed to present for scheduled appointments).

### Clinical Outcomes

**Figure 3:**
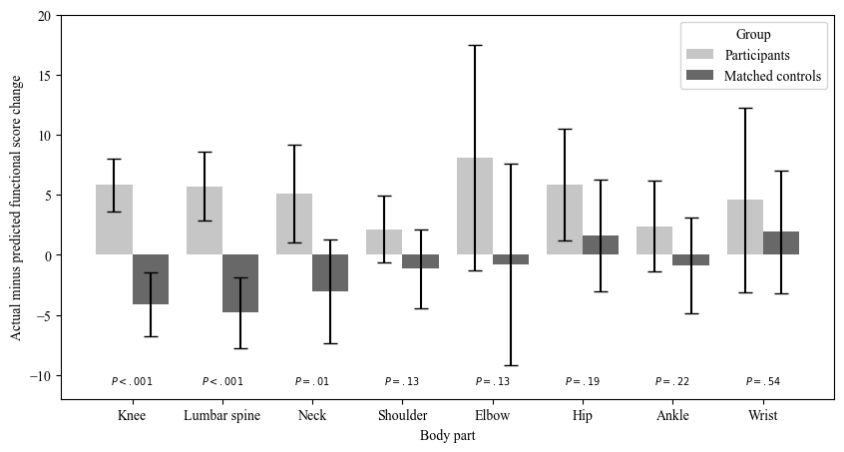
Change in functional status (actual minus predicted) for participants and matched controls by body part.

Participants in TPT within an advanced primary care model achieved significantly greater improvements in functional status compared to FOTO controls and required fewer clinic visits than control patients (Figure 3). On average, FOTO functional status residual (which is the actual functional status change minus the predicted functional status change) was 5.82 (*P*<.001) for patients with knee pain, 5.70 (*P*<.001) for patients with lumbar spine pain, and 5.07 (*P*=.007) for patients with neck pain. Other body parts, such as the thoracic spine, shoulder, elbow, foot, pelvis, ankle, wrist, leg, and arm, also showed more improvement in functional score throughout treatment compared to FOTO controls, albeit not statistically significantly (Figure 3). The functional status residual (difference between what was observed for TPT patients compared to FOTO predicted) was –4.14 for knee pain, –4.80 for lumbar spine pain, and –3.06 for neck pain. In the FOTO control population, participants generally had less functional clinical improvement than predicted. This demonstrates that patients in TPT integrated in advanced primary care had statistically and clinically significant improvements in their functional status compared with FOTO controls.

**Figure 4:**
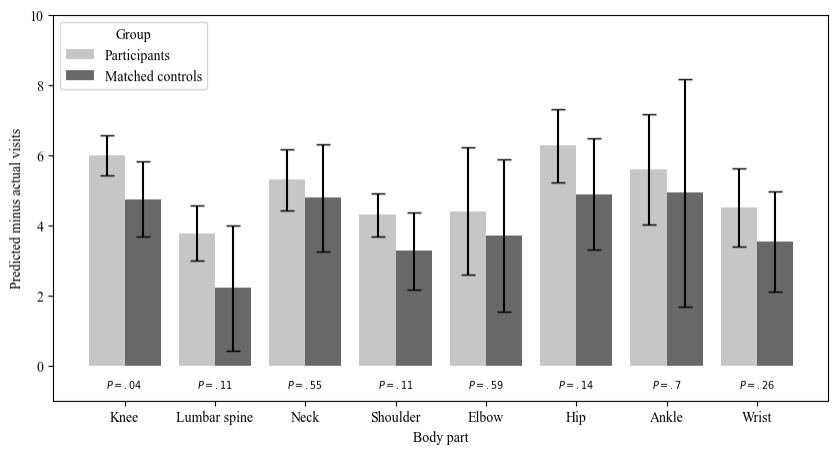
Difference in the number of visits required to achieve improvement in functional status (predicted minus actual number of visits) for participants and controls.

On average, TPT patients required 5.41 (SD 2.66) visits to resolution of symptoms. This compares favorably to the FOTO risk-adjusted predicted number of visits, which was 10.3 (SD 1.55), and to the national benchmark from the FOTO matched controls of 6.49 (SD 5.47 visits; Figure 4).

### Provider Reported Patient Progress

Providers reported patient progress for 473 patients. Recovery, defined as patients either meeting, mostly meeting, or on track to meet expectations, was achieved for 461 (97.5%) of participants. Breakdown of these categories are as follows: meeting expectations (68/461, 14.8%), mostly meeting expectations (4/461, 0.9%), and on track to meet expectations (389/461, 84.4%).

### Patient Satisfaction

Overall participant satisfaction was high, with a net promoter score of 97 for PTs and a response rate of 22%.

### Costs of TPT

Direct cost of PT care in 2023 for patients receiving care in California ranged from US $176 to US $288 per visit. Since the results of this study found that the intervention reduced the total number of PT visits by 4.9 when using the FOTO database prediction and by 1.1 when using the FOTO control, we estimate that the total cost savings for this intervention ranges from US $193 to US $1411 per injury. Related cost savings, such as procedure, imaging, and surgery reduction, were not included.

## Discussion

### Principal Results

This study of a TPT intervention integrated with primary care for a demographically diverse population demonstrated 4 key findings. First, 1257/1375 (91.4%) participants who were prescribed and completed TPT had successful treatment of their musculoskeletal issue. This highlights that a TPT approach, when integrated into primary care and triaged by a PCP, is appropriate for most patients.

Second, patients achieved clinically significant outcomes in fewer visits than controls. TPT patients required 5.4 visits to symptom resolution, compared to 6.5 visits for controls and 10.3 predicted visits from risk-adjusted benchmarks. Several components of the studied model likely contribute to efficient care, including easy access to PT, guideline-driven care by both primary care providers and physical therapists, and care collaboration among providers. Additionally, when PT is delivered through videoconferencing, self-management techniques (eg, self-massage) may take the place of passive, time-consuming treatments, such as manual therapy [33].

Third, knee pain, lumbar spine pain, and neck pain had the most statistically significant clinical improvement compared to FOTO controls. Other body parts showed similarly large average improvements, such as the foot, elbow, thoracic spine, pelvis, hip, and upper leg. However, we lacked sufficient participants in these categories to show statistical significance. These findings can perhaps be explained by the fact that the knee and back respond well to education and exercises that do not require equipment, both of which can be delivered through videoconferencing [20]. Early and ongoing studies of the knee, specifically TPT post knee arthroplasty, have consistently shown value [34,35]. In contrast, shoulder pain, especially given the prevalence of adhesive capsulitis, often has a long recovery period and can take up to 2 years to resolve [36].

Fourth, given the reduction in required PT visits to achieve patients’ clinical goals, the TPT intervention saved an estimated US $193 to US $1411 per injury. This is a conservative estimate since it does not include any savings from avoided imaging or specialist visits.

### Comparisons to Prior Work

The findings of this study align with prior evaluations demonstrating that early access to PT is associated with improved outcomes with fewer PT visits [8-12] and that patients receiving TPT can achieve excellent functional status gains [23-25]. Other authors have found that TPT offerings typically lower the cost of delivering PT and may improve adherence and speed up the initiation of therapy, resulting in lower average health care spending across the population of people with musculoskeletal disorders [37,38]. TPT options may be particularly appropriate for patients with lower-acuity musculoskeletal issues [14] and appealing for people who cannot easily reach in-person PT clinics because of transportation issues, mobility limitations, or geographic access barriers [39,40].

Multiple studies have documented that musculoskeletal education is underrepresented in primary care training, resulting in lower knowledge scores, reduced clinical confidence, and suboptimal management of patients with musculoskeletal concerns [41-43]. The primary care providers in the model evaluated received considerable musculoskeletal training and ongoing support, requesting nearly three peer-to-peer musculoskeletal expert opinion consults per week. The benefit of this close collaboration between primary care and musculoskeletal experts in the primary care setting benefits patients. Prior studies have demonstrated that patient confidence is increased when musculoskeletal diagnoses and care are discussed in an integrated manner across multiple providers [32]. Beyond the PT-specific care, primary care integration enables treatment of both the musculoskeletal condition and the comorbid conditions in tandem (eg, obesity, anxiety, and depression), which prior studies suggest may contribute to more efficient recovery [25,33].

### Future Work

Our results suggest 3 key areas for future work. First, given the prevalence of mental health–related comorbidities, such as depression and anxiety, among patients with musculoskeletal complaints [44], it would be worthwhile to measure mental health–related outcomes, both at baseline and to understand how they change with improvements in functional status. Second, given historically poor access to health care services among vulnerable populations (eg, rural, lower income, poor technology access, or literacy), it will be important for future work to specifically evaluate the implementation of an integrated musculoskeletal offering, such as the one studied here with these groups. Third, the promising savings finding warrants a more comprehensive economic evaluation that includes costs beyond reduced PT visits, such as costs for prevented imaging, specialist visits, and musculoskeletal interventions, which were not included in this analysis. The most recent analysis accounting for both the direct and indirect costs of PT treatment was published by the American Physical Therapy Association [45], with net benefit calculations for musculoskeletal conditions ranging from US $4160 for low back pain to US $39,533 for carpal tunnel syndrome.

### Limitations

This study had 5 key limitations. First, this study focused on commercially insured populations. Given the promising results, it warrants future evaluations in older (Medicare) populations. Second, the patients were largely concentrated in California. Although the One Medical integrated care model operates nationwide, and we do not anticipate significant differences in other geographies, this warrants validation in other locations with different labor costs and reimbursement rates. Third, we excluded patients who had only a single PT visit, both because we sought to evaluate their change in functional status over time and because most patients requiring outpatient PT need an initial assessment followed by a course of care [46]. However, this may imply that the findings are not generalizable to populations requiring only a single PT visit. Fourth, the cost outcomes were estimated from One Medical claims for in-person PT. An analysis of all costs for patients receiving TPT in advanced primary care systems should be a priority. Finally, only 22.3% (348/1563) of participants responded to the patient satisfaction survey, and although they provided highly favorable feedback, future evaluations should prioritize the comprehensive collection of patient satisfaction and experience information.

### Conclusions

Given the key finding that participants in TPT—when patient-centric and embedded in primary care—were able to achieve recovery of their musculoskeletal issues in fewer visits and with greater functional improvement than controls, this model holds promise for addressing the escalating musculoskeletal costs of the US commercially insured population.

## Appendix

Multimedia Appendix 1 Primary care provider educational content embedded in the electronic health record: Example for low back pain.

Multimedia Appendix 2 Ready-to-prescribe at-home treatment program content for patients: Example for shoulder pain .

## Abbreviations

FOTO: Focus on Therapeutic Outcomes
PCP: primary care physician
PT: physical therapy
TPT: tele-physical therapy

## Data Availability

Summary data will be made available upon reasonable request from the corresponding author.

## References

[1] (2023). Workforce health index: 2023. apree health.

[2] Lawrence RC, Felson DT, Helmick CG, Arnold LM, Choi H, Deyo RA, Gabriel S, Hirsch R, Hochberg MC, Hunder GG, Jordan JM, Katz JN, Kremers HM, Wolfe F, National Arthritis Data Workgroup (2008). Estimates of the prevalence of arthritis and other rheumatic conditions in the United States. Part II. Arthritis Rheum.

[3] Dieleman JL, Cao J, Chapin A, Chen C, Li Z, Liu A, Horst C, Kaldjian A, Matyasz T, Scott KW, Bui AL, Campbell M, Duber HC, Dunn AC, Flaxman AD, Fitzmaurice C, Naghavi M, Sadat N, Shieh P, Squires E, Yeung K, Murray CJL (2020). US health care spending by payer and health condition, 1996-2016. JAMA.

[4] Bryla J (2013). Low back pain takes toll on worker health and productivity. Integrated Benefits Institution.

[5] Greggi C, Visconti V, Albanese M, Gasperini B, Chiavoghilefu A, Prezioso C, Persechino B, Iavicoli S, Gasbarra E, Iundusi R, Tarantino U (2024). Work-related musculoskeletal disorders: a systematic review and meta-analysis. J Clin Med.

[6] Manning C, Jorgensen M (2024). The price of pain: workers compensation costs for musculoskeletal claims in the state of Kansas, 2014-2022. J Occup Environ Med.

[7] Bevan S (2015). Economic impact of musculoskeletal disorders (MSDs) on work in Europe. Best Pract Res Clin Rheumatol.

[8] Zigenfus GC, Yin J, Giang GM, Fogarty WT (2000). Effectiveness of early physical therapy in the treatment of acute low back musculoskeletal disorders. J Occup Environ Med.

[9] Hon S, Ritter R, Allen D (2021). Cost-effectiveness and outcomes of direct access to physical therapy for musculoskeletal disorders compared to physician-first access in the United States: systematic review and meta-analysis. Phys Ther.

[10] Ojha H, Wyrsta N, Davenport T, Egan W, Gellhorn A (2016). Timing of physical therapy initiation for nonsurgical management of musculoskeletal disorders and effects on patient outcomes: a systematic review. J Orthop Sports Phys Ther.

[11] Marrache M, Prasad N, Margalit A, Nayar SK, Best MJ, Fritz JM, Skolasky RL (2022). Initial presentation for acute low back pain: is early physical therapy associated with healthcare utilization and spending? A retrospective review of a national database. BMC Health Serv Res.

[12] Babatunde OO, Bishop A, Cottrell E, Jordan JL, Corp N, Humphries K, Hadley-Barrows T, Huntley AL, van der Windt DA (2020). A systematic review and evidence synthesis of non-medical triage, self-referral and direct access services for patients with musculoskeletal pain. PLoS One.

[13] (2020). Large employers’ health care strategy and plan design survey. Business Group on Health.

[14] (2024). Completed assessment: virtual musculoskeletal (MSK) solutions. Peterson Health Technology Institute.

[15] (2020). Musculoskeletal facets. Business Group on Health.

[16] Lord D, Wright J, Fung R, Lederhaus E, Taylor K, Watts S, Hagg H, Bravata D (2019). Integrated physical medicine at employer-sponsored health clinics improves quality of care at reduced cost. J Occup Environ Med.

[17] Madhusudhan DK, Watts SA, Lord DJ, Ding F, Lawrence DC, Sheldon A, Leonard J, Bravata DM (2021). Employer-sponsored health centers provide access to integrated care via a hybrid of virtual and in-person visits. Telemed Rep.

[18] Cantor JH, McBain RK, Pera MF, Bravata DM, Whaley CM (2021). Who is (and Is Not) receiving telemedicine care during the COVID-19 pandemic. Am J Prev Med.

[19] Horn ME, Fritz JM (2018). Timing of physical therapy consultation on 1-year healthcare utilization and costs in patients seeking care for neck pain: a retrospective cohort. BMC Health Serv Res.

[20] Arnold E, La Barrie J, DaSilva L, Patti M, Goode A, Clewley D (2019). The effect of timing of physical therapy for acute low back pain on health services utilization: a systematic review. Arch Phys Med Rehabil.

[21] Fatoye F, Gebrye T, Mbada C, Useh U (2023). Economic evaluations of digital health interventions for the management of musculoskeletal disorders: systematic review and meta-analysis. J Med Internet Res.

[22] McKay SE, Buono FD, Walker J, Glinski C, Printz DMB, Brienza R (2021). Impact of interprofessional embedding of physical therapy in a primary care training clinic. J Interprof Care.

[23] Corso M, Cancelliere C, Mior S, Salmi LR, Cedraschi C, Nordin M, Sci DM, Taylor-Vaisey A, Côté P (2022). Are nonpharmacologic interventions delivered through synchronous telehealth as effective and safe as in-person Interventions for the management of patients with nonacute musculoskeletal conditions? A systematic rapid review. Arch Phys Med Rehabil.

[24] Bargeri S, Castellini G, Vitale J, Guida S, Banfi G, Gianola S, Pennestrì F (2024). Effectiveness of telemedicine for musculoskeletal disorders: umbrella review. J Med Internet Res.

[25] Hewitt S, Sephton R, Yeowell G (2020). The effectiveness of digital health interventions in the management of musculoskeletal conditions: systematic literature review. J Med Internet Res.

[26] Gozalo PL, Resnik LJ, Silver B (2016). Benchmarking outpatient rehabilitation clinics using functional status outcomes. Health Serv Res.

[27] Bekmuratova S, Bahle-Lampe A, Pflaster T (2023). Physical therapists' experience using focus on therapeutic outcome in outpatient clinics: a qualitative study. Health Serv Manage Res.

[28] Edmond S, Werneke M, Grigsby D, Young M, Harris G (2023). The association between self-efficacy on function and pain outcomes among patients with chronic low back pain managed using the McKenzie approach: a prospective cohort study. J Man Manip Ther.

[29] (2023). What definitions are used by FOTO to define FOTO outcome measurement scores?. FOTO Patients Outcomes.

[30] (2023). One Medical.

[31] Stewart M, Loftus S (2018). Sticks and stones: the impact of language in musculoskeletal rehabilitation. J Orthop Sports Phys Ther.

[32] O'Connor MI, Chudy C, Peters KC, Ribaudo M, McCulloch C, Aguilar J, Taylor T, Grant RA (2025). Patients' experience with evaluation by both a musculoskeletal physician and physical therapist in the same digital visit: survey study. JMIR Form Res.

[33] Valentijn PP, Tymchenko L, Jacobson T, Kromann J, Biermann CW, AlMoslemany MA, Arends RY (2022). Digital health interventions for musculoskeletal pain conditions: systematic review and meta-analysis of randomized controlled trials. J Med Internet Res.

[34] Prvu Bettger J, Green CL, Holmes DN, Chokshi A, Mather RC, Hoch BT, de Leon AJ, Aluisio F, Seyler TM, Del Gaizo DJ, Chiavetta J, Webb L, Miller V, Smith JM, Peterson ED (2020). Effects of virtual exercise rehabilitation in-home therapy compared with traditional care after total knee arthroplasty: VERITAS, a randomized controlled trial. J Bone Joint Surg Am.

[35] Charalambous A, Ekhtiari S, Wainwright A, Najafi R, Chaudhry H, Pincus D, Ravi B (2024). Virtual versus in-person physiotherapy following total knee arthroplasty: a comparative analysis. Int Orthop.

[36] Le HV, Lee SJ, Nazarian A, Rodriguez EK (2017). Adhesive capsulitis of the shoulder: review of pathophysiology and current clinical treatments. Shoulder Elbow.

[37] Napoleone J, Devaraj S, Noble M, Parrinello C, Jasik C, Norwood T, Livingstone I, Linke S (2025). Health care cost savings and utilization reductions associated with virtual physical therapy care: a propensity-matched claims analysis. Phys Ther.

[38] Hayes AJ, Withers HG, Glinsky JV, Chu J, Jennings MD, Starkey I, Parmeter R, Boulos M, Cruwys JJ, Duong K, Jordan I, Wong D, Trang S, Duong M, Liu H, Lambert TE, Zadro JR, Sherrington C, Maher C, Lucas BR, Taylor D, Ferreira ML, Harvey LA (2025). Remotely delivered physiotherapy for musculoskeletal conditions is cost saving for the health system and patients: economic evaluation of the REFORM randomised trial. J Physiother.

[39] Lee AC, Deutsch JE, Holdsworth L, Kaplan SL, Kosakowski H, Latz R, McNeary LL, O'Neil J, Ronzio O, Sanders K, Sigmund-Gaines M, Wiley M, Russell T (2024). Telerehabilitation in physical therapist practice: a clinical practice guideline from the American physical therapy association. Phys Ther.

[40] Hawley-Hague H, Lasrado R, Martinez E, Stanmore E, Tyson S (2023). A scoping review of the feasibility, acceptability, and effects of physiotherapy delivered remotely. Disabil Rehabil.

[41] DiGiovanni B, Sundem L, Southgate R, Lambert D (2016). Musculoskeletal medicine is underrepresented in the American medical school clinical curriculum. Clin Orthop Relat Res.

[42] Lynch JR, Schmale GA, Schaad DC, Leopold SS (2006). Important demographic variables impact the musculoskeletal knowledge and confidence of academic primary care physicians. J Bone Joint Surg Am.

[43] Goff I, Wise E, Coady D, Walker D (2016). Musculoskeletal training: are GP trainees exposed to the right case mix for independent practice?. Clin Rheumatol.

[44] Bair MJ, Wu J, Damush TM, Sutherland JM, Kroenke K (2008). Association of depression and anxiety alone and in combination with chronic musculoskeletal pain in primary care patients. Psychosom Med.

[45] (2023). The economic value of physical therapy in the United States. American Physical Therapy Association.

[46] Machlin SR, Chevan J, Yu WW, Zodet MW (2011). Determinants of utilization and expenditures for episodes of ambulatory physical therapy among adults. Phys Ther.

