# Supplementary material for: Clinical and Economic Outcomes Associated With Musculoskeletal Care in an Integrated Advanced Primary Care Model: Controlled Cohort Analysis
Source: J Med Internet Res. 2025 Oct 7;27:e76794. doi: 10.2196/76794 (PMC12541268; doi:10.2196/76794)
Supplement: Multimedia Appendix 1 [file jmir_v27i1e76794_app1.docx]

# Figure S1. Primary care provider educational content embedded in the electronic health record: Example for low back pain.

#
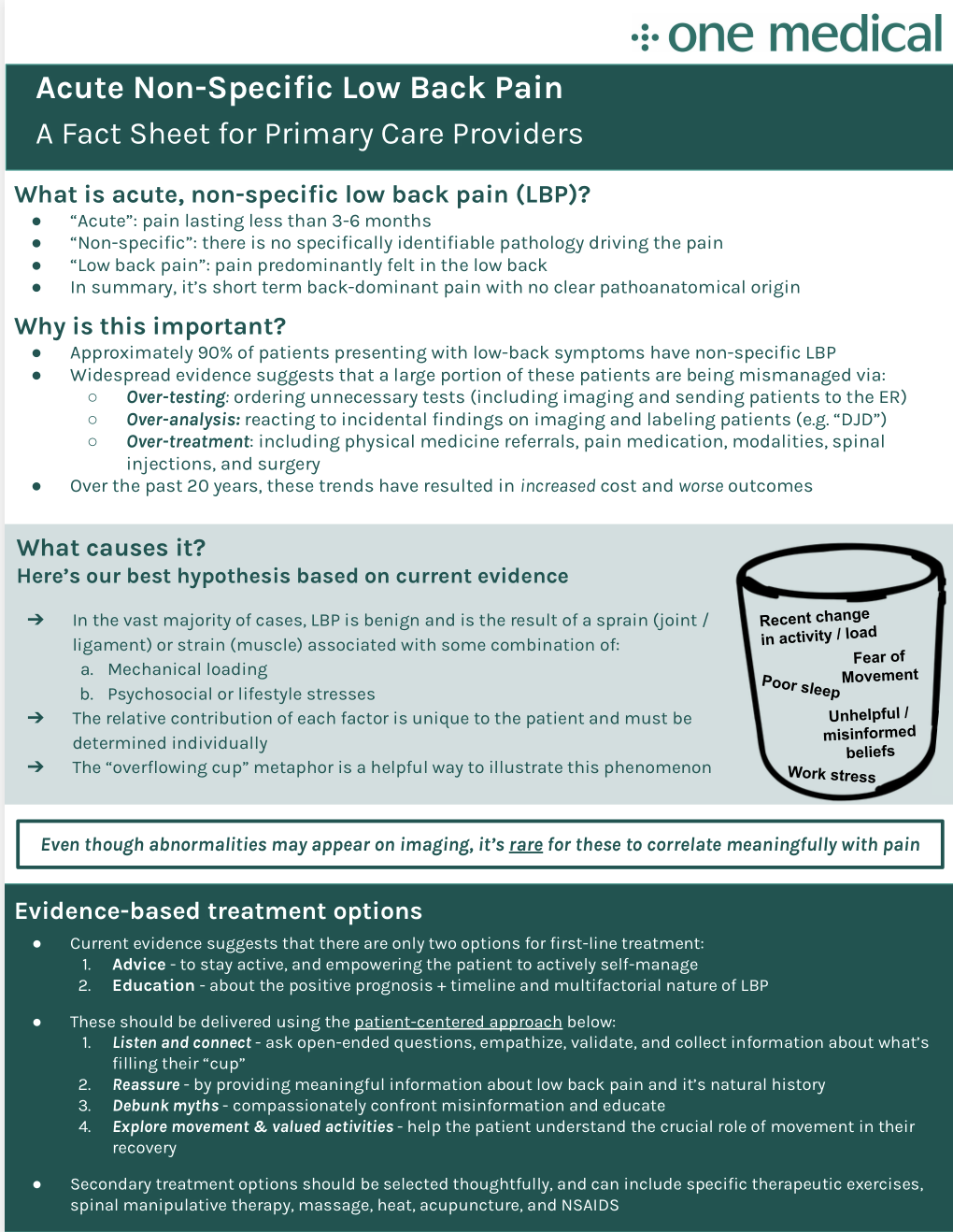


#
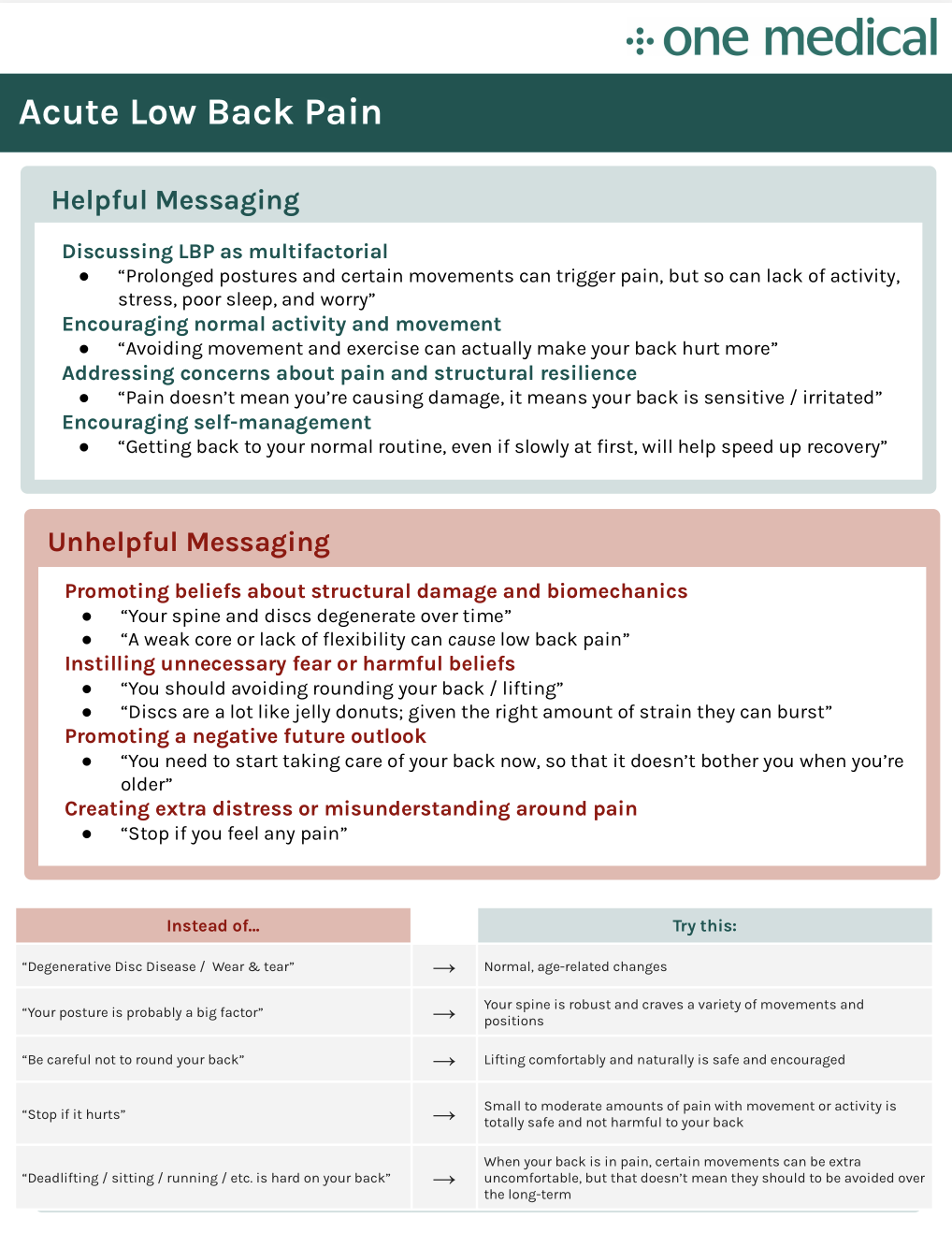


# 
