# Supplementary material for: Clinical and Economic Outcomes Associated With Musculoskeletal Care in an Integrated Advanced Primary Care Model: Controlled Cohort Analysis
Source: J Med Internet Res. 2025 Oct 7;27:e76794. doi: 10.2196/76794 (PMC12541268; doi:10.2196/76794)

# Figure S2. Ready-to-prescribe at-home treatment program content for patients: Example for shoulder pain.


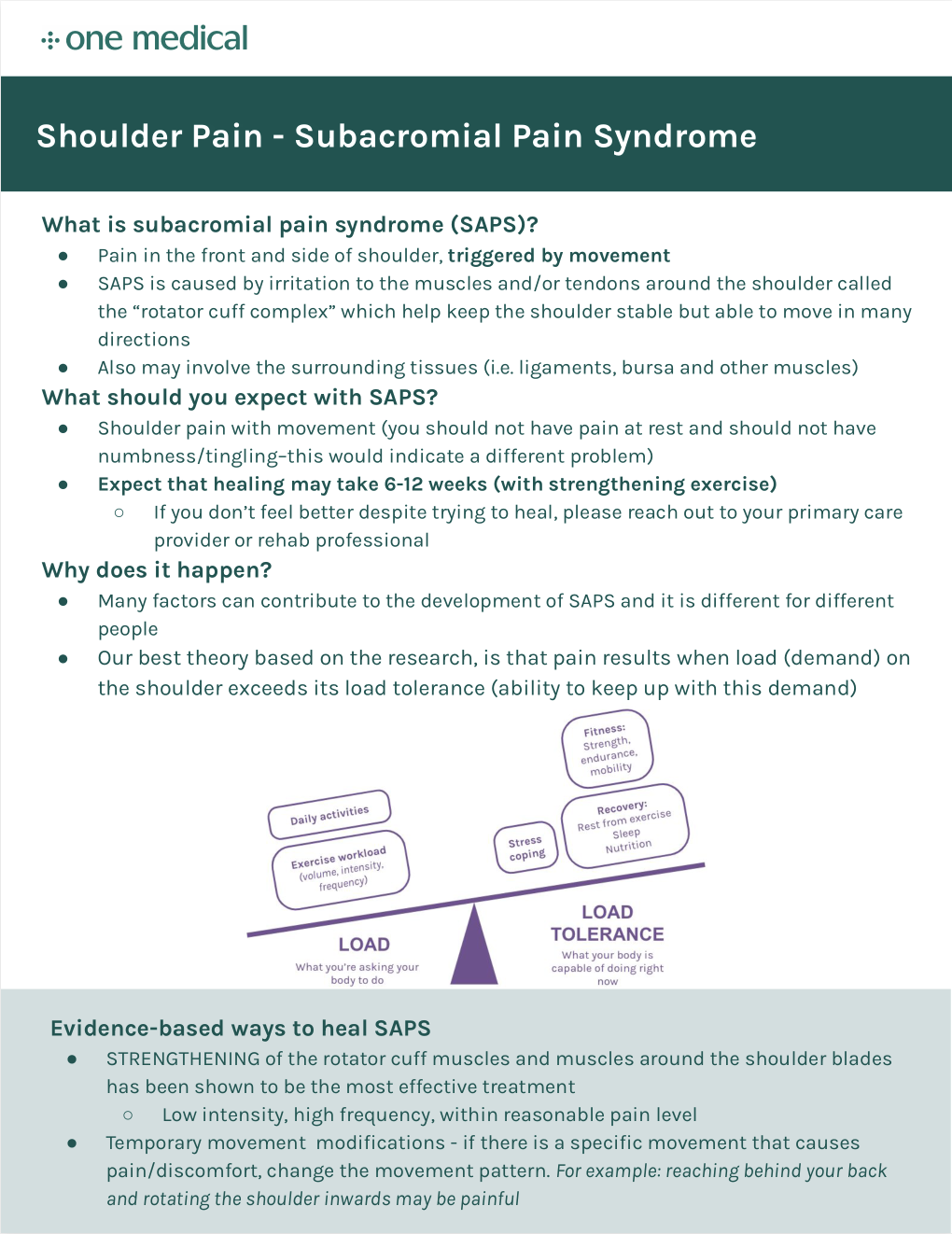


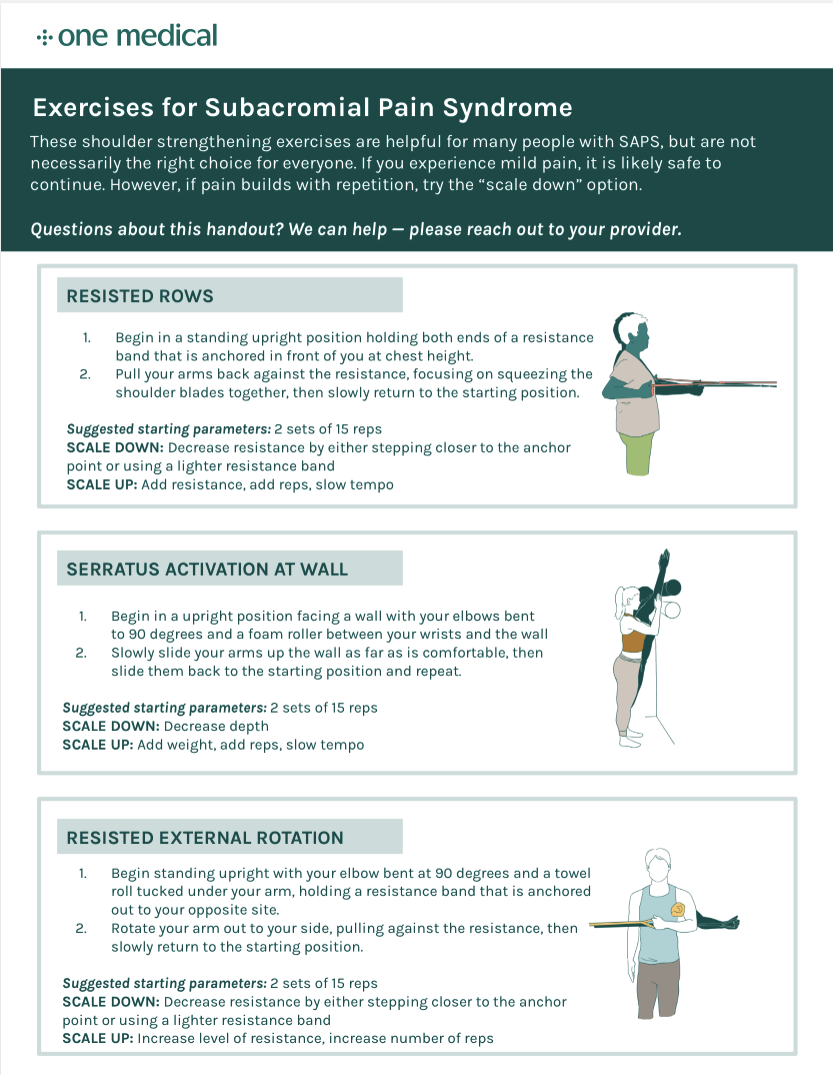

Supplement: Multimedia Appendix 2 [file jmir_v27i1e76794_app2.docx]
